# Effects of sourdough and yeast fermented breads consumption on glycaemia in healthy populations: a systematic review

**DOI:** 10.3389/fnut.2026.1910648

**Published:** 2026-08-04

**Authors:** Malgorzata Starowicz, Julieta Domínguez-Soberanes, Enriqueta Garcia-Gutierrez, Sultan Arslan-Tontul, Kathryn J. Burton-Pimentel, Bojana Vidović, Isabel Moreno-Indias, Mustafa Fevzi Karagöz, Milica Prvulović, Valentina Simeunovic, Patricia Ruiz-Limón, Carolina Gutiérrez-Repiso, Zada Pajalic, Oktay Yerlikaya, Tuba Esatbeyoglu, Birsen Yilmaz, Fani Mantzouridou, Christophe Chassard, Smilja Pracer, Guy Vergères, Sandrine Louis

**Affiliations:** 1 Chemistry and Biodynamics of Food Team, InLife Institute of Animal Reproduction and Food Research, Polish Academy of Sciences, Olsztyn, Poland; 2 Facultad de Ingeniería, Universidad Panamericana, Mexico City, Mexico; 3 Department of Agronomic Engineering, Technical University of Cartagena, Cartagena, Spain; 4 Food Engineering Department, Agricultural Faculty, Selcuk University, Konya, Türkiye; 5 Microbial Food Systems Research Division, Agroscope, Bern, Switzerland; 6 Department of Bromatology, Faculty of Pharmacy, University of Belgrade, Belgrade, Serbia; 7 Instituto de Investigación Biomédica de Málaga y Plataforma en Nanomedicina-IBIMA Plataforma BIONAND, Málaga, Spain; 8 Department of Endocrinology and Nutrition, Virgen de la Victoria University Hospital, Málaga, Spain; 9 Centro de Investigación Biomédica en Red de la Fisiopatología de la Obesidad y Nutrición (CIBEROBN), Instituto de Salud Carlos III, Madrid, Spain; 10 Nutrition and Dietetics Department, Health Sciences Institute, Hitit University, Çorum, Türkiye; 11 Institute for Biological Research “Siniša Stanković”, National Institute of the Republic of Serbia, University of Belgrade, Belgrade, Serbia; 12 Faculty of Health and Social Sciences, University of South-Eastern Norway (USN), Kongsberg, Norway; 13 Department of Dairy Technology, Faculty of Agriculture, Ege University, Bornova, Türkiye; 14 Department of Molecular Food Chemistry and Food Development, Institute of Food and One Health, Gottfried Wilhelm Leibniz University Hannover, Hanover, Germany; 15 Department of Biological Sciences, Tata Institute of Fundamental Research, Hyderabad, India; 16 Laboratory of Food Chemistry and Technology, School of Chemistry, Aristotle University of Thessaloniki, Thessaloniki, Greece; 17 UCA, INRAE, VetAgro Sup, UMRF, Aurillac, France; 18 Laboratory for Molecular Neurobiology and Behavior, Institute for Biological Research “Sinisa Stankovic”, University of Belgrade, Belgrade, Serbia; 19 Institute of Nutritional and Food Microbiology, Max Rubner-Institut, Karlsruhe, Germany

**Keywords:** bread, fermentation, glucose response, insulin sensitivity, sourdough, systematic review

## Abstract

Bread is a vital source of energy and is widely consumed in human diets. As a carbohydrate-rich food, it is often considered to impair glycaemic homeostasis; however, clinical and molecular evidence suggest that its effects may vary depending on the characteristics of bread consumed. In particular, sourdough-fermented bread has been proposed to exert potentially beneficial effects on glycaemia via shifts in metabolites in the food matrix, as well as chemical and physical changes that occur during fermentation. However, evidence from human interventional studies on this topic is inconsistent due to differing experimental designs, heterogeneous populations, or a focus on sourdough fermentation that does not consider the effects of yeast fermentation. Our objective was to evaluate the evidence for a positive effect of fermented bread consumption on glycaemia and insulinaemia in healthy adults. To this end, we conducted a systematic review to identify and analyze relevant human studies, complemented by a characterization of the breads investigated and a discussion of possible mechanisms of action, following the EFSA dossier framework. We identified 27 studies that met our inclusion criteria. Taking into account the risk of bias in these studies, the consistency of the reported effects, and the biological plausibility based on food characteristics and potential mechanisms of action, we conclude that the available evidence for a beneficial effect of sourdough compared to yeast-fermented bread on glycaemic and insulinaemic homeostasis in healthy individuals is neither sufficient nor convincing. For the general effect of fermentation on glycaemic and insulinaemic homeostasis in healthy individuals, evidence is considered very low.

## 1 Introduction

Fermented foods (FFs) constitute a diverse and ancient category of food products that are produced via the controlled activity of microorganisms (bacteria, yeasts, or molds), acting on raw food materials of both plant and animal origins (including cereals, legumes, vegetables, roots and tubers, milk, meat, or fish). Defined as “foods made through desired microbial growth and enzymatic conversions of food components” (1), FFs have been used in human cultures worldwide for millennia, extending shelf-life, influencing flavors, aromas, textures and modifying the nutritional and functional characteristics of foods. More recently, FFs have also been recognized for their potential roles in modulating the gut microbiota, supporting metabolic health and contributing to the prevention of chronic diseases, including type 2 diabetes mellitus (T2D) (2).

The increasing prevalence of T2D represents one of the most critical public health challenges of the 21st century, placing a considerable burden on healthcare systems worldwide (3, 4). T2D is characterized by chronic hyperglycaemia resulting from impaired insulin secretion and action. The condition is driven by a complex interplay of genetic, metabolic, and lifestyle factors, with dietary factors playing an important role in the disease pathogenesis and development. According to the World Health Organization, T2D is diagnosed by elevated fasting plasma glucose or abnormal glucose tolerance (5). As rates of T2D continue to rise globally, substantial research has been invested in identifying dietary strategies to prevent or delay disease onset among healthy individuals and particularly among populations at increased risk of T2D, such as those with prediabetes or obesity. Carbohydrate quality and consumption are important dietary components implicated in T2D risk (6, 7) and its management (8, 9). Of the different dietary sources of carbohydrates, bread is widely understood to induce rapid rises in glycaemia, and indeed, white bread is often used as the reference (in place of glucose) for assessing glycaemic index (GI), a measure indicating the rise of blood glucose in the 2 h following consumption of the food (10).

In this context, understanding the glycaemic impact of different breads, particularly in relation to processing and fermentation methods, is essential for dietary management. The practice of fermenting grains to produce bread has a long history, with both baker’s yeast and sourdough breads consumed across cultures for millennia. While some breads are processed without a fermentation step (e.g., soda bread), bread fermentation is common, improving sensory properties, shelf-life (11, 12), can increase the bio-accessibility of nutrients (11, 13), and generate bioactive compounds that may alter carbohydrate structure in ways that may positively affect postprandial metabolism (11, 14). A number of systematic reviews have already considered the growing body of evidence on the effects of different types of fermented bread on glycaemic control and insulin sensitivity (14–16). However, these previous reviews often pooled broad study populations and diverse bread types with different cereal or fiber contents. Such an approach prevents solid conclusions about healthy participants and makes it difficult to elucidate the role of fermentation in the observed effects. To our knowledge, no previous review has explicitly evaluated the effect of fermentation and fermentation type in intervention studies with a focus on healthy populations.

The present review addresses these challenges by characterizing bread, its constituents, and modes of production, and by examining how these might explain purported effects on glucose homeostasis. It uses a systematic approach to identify relevant clinical trials investigating the effects of fermented bread on glycaemia and reports on possible mechanisms of action underlying the observed effects. Using key outcomes of glucose homeostasis such as fasting blood glucose, insulin sensitivity, postprandial glucose, and insulin responses, we sought to determine whether regular consumption of fermented breads can support metabolic health in healthy adults, including those with obesity and pre-diabetes, to reflect populations at risk of T2D. Within this overarching aim, we examined three specific research questions: Does the type of fermentation influence the effects of bread on glycaemia (i.e., mainly sourdough versus yeast-based fermentation)? Does fermentation impact the glycaemic effects (i.e., fermented breads versus non-fermented breads or other kinds of cereal-based food)? Does the consumption of specific breads affect glycaemic parameters, considering both differences between low and high intakes and changes between pre- and post-intervention? Through this comprehensive and systematic synthesis, we intend to clarify whether fermented breads offer measurable benefits for glucose regulation in healthy adults and to provide evidence that may inform both clinical nutrition practice and public dietary recommendations.

## 2 Methods

This review was conducted following a generic search strategy, developed within the COST Action CA20218 Promoting Innovation of fermented foods PIMENTO initiative, as detailed in a corresponding position paper (17), and in agreement with the EFSA guidelines (18, 19). This review is structured in three main parts: (i) a systematic review of human studies in accordance with published guidelines (20, 21) (see Supplementary file 1 for a PRISMA checklist), as well as (ii) complementary parts on food characterization and (iii) mechanism of action. The review protocol, including specific search terms and strategy, was registered in the Open Science Framework (OSF protocol, https://doi.org/10.17605/OSF.IO/QRZA3).

The EFSA framework for the scientific substantiation of health claims (Regulation (EC) No 1924/2006) requires that a claimed effect be evaluated on the basis of (i) a sufficiently characterized food or food constituent; (ii) a defined beneficial physiological effect demonstrated in human intervention studies of adequate quality and consistency; and (iii) a biologically plausible mechanism of action supporting the observed effect. Our review follows this framework and evaluates whether the current body of evidence on fermented bread consumption meets these three requirements for a claim related to glycaemic and insulinaemic homeostasis in healthy adults.

### 2.1 Systematic review of human studies

#### 2.1.1 Literature search

**Figure 1:**
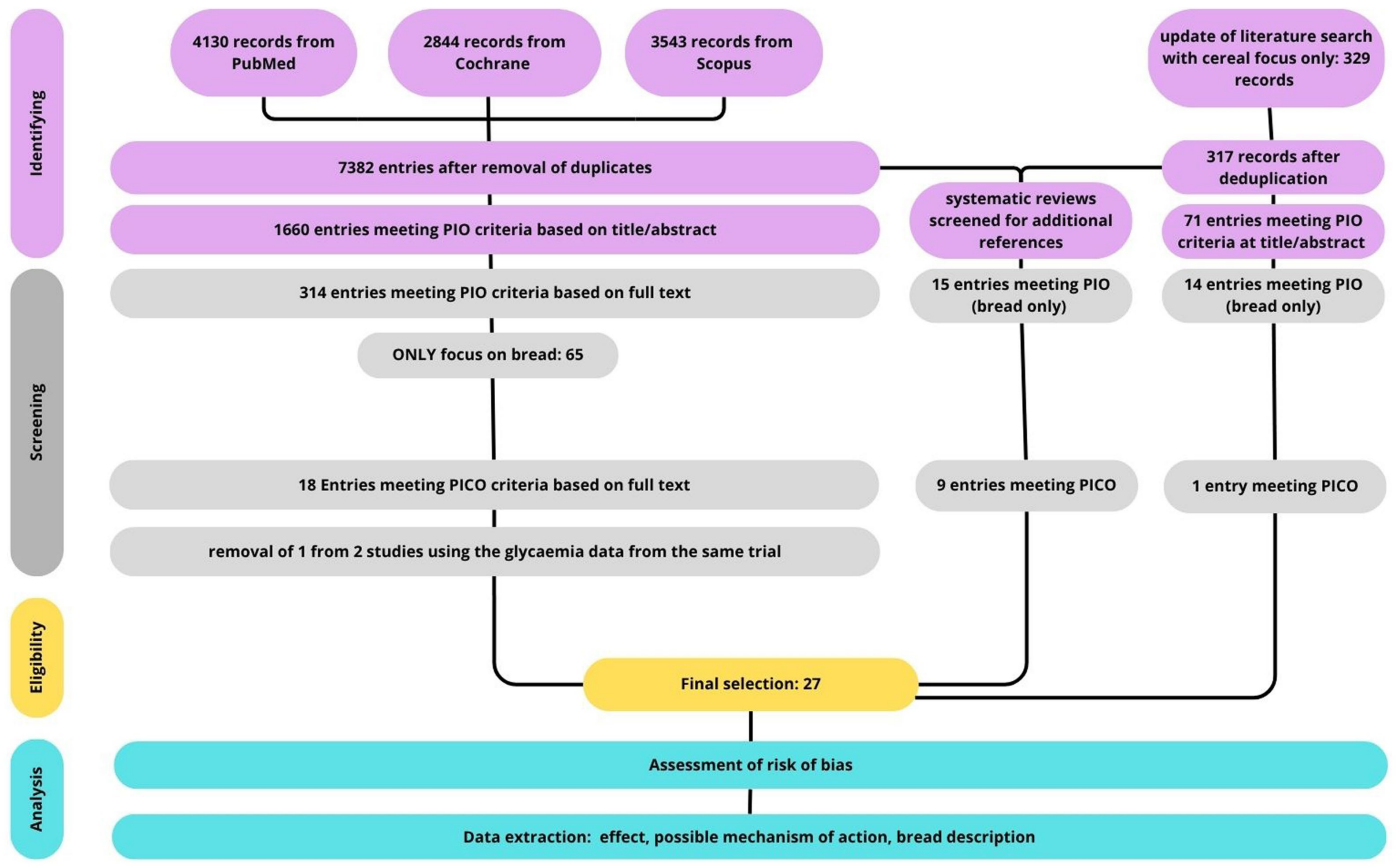
Flow chart showing the methodology and results of study selection.

A literature search was conducted using Medline, Cochrane, and Scopus. The search was limited to English-language studies and used predefined strings (see Supplementary file 2). The studies included in this review were identified through (i) selection of articles that met the predefined eligibility criteria aligned with the P/I/C/O (Population, Intervention, Control, Outcome) framework (no exclusion criteria on study design), (ii) screening of relevant systematic reviews for possibly missed studies meeting the same criteria, as shown in the flow diagram (Figure 1). From 7,382 initial records (post-deduplication), 1,660 met PIO (Population/Intervention/ Outcome) criteria based on title and abstract. After full-text assessment and application of refined inclusion criteria, 314 entries were retained.

The selection was further narrowed to 65 articles focused on bread as the fermented product of interest. Of these, 18 included an appropriate control, but 2 used exactly the same data from a single trial; one was excluded, and 17 were retained for this review.

An update of the search (from September 2023 to January 2025), focusing on fermented cereals only, retrieved 329 new entries (317 after deduplication), resulting in 14 full-text eligible studies and 1 meeting full PICO (Population/Intervention/Control/Outcome) criteria.

Review articles were retained at the screening stage if they addressed related topics, allowing for the identification of missed pertinent primary studies. Systematic reviews identified during the search contributed 15 entries meeting the PIO framework; 9 met the full PICO criteria and were included.

#### 2.1.2 Selection criteria

Eligibility according to the PIO criteria was assessed using CADIMA, an open-source platform designed to support systematic literature reviews and evidence mapping (22). Two reviewers screened the studies independently and resolved discrepancies through discussion, involving a third reviewer in the event of a conflict. Inclusion was limited to original studies involving at least in part healthy or pre-diabetic adults, but not athletes or pregnant women. We defined “healthy” as without a diagnosed disease and without regular use of medications. We however made exceptions for contraceptive pills, postmenopausal hormone treatment, and well-adjusted thyroid hormone therapy. Studies measuring blood glucose or insulin homeostasis were selected.

The intervention or exposure was initially defined as the oral intake of any type of FF, or as instances where FF consumption could be inferred from dietary questionnaires. Interventions were excluded if FFs were mixed with other components that prevented the effect of the FF from being isolated/assessed independently. Also, studies testing only probiotics (without FF matrix, or without a direct participation in the fermentation of the food matrix) or only an extract from the FF were excluded, as we were interested in the effect of the whole food. Non-oral applications were not considered. Outcomes of interest were defined as measures related to glucose or insulin homeostasis, or, at a minimum, the collection of relevant blood samples indicative of metabolic responses. In a second step, we focused our search on studies assessing bread. Eligible controls for fermented breads were defined as non-fermented cereal products (e.g., pasta, porridge, unfermented breads), provided that they used the same cereal and flour type. Comparisons of leavening methods, such as yeast-fermented vs. sourdough breads, were also eligible, provided breads were produced using similar flour/cereal. “Long-term” intervention studies that offered a before/after comparison were accepted in the absence of a proper control. Studies that only compared breads to glucose drinks were excluded. Observational studies employing dietary questionnaires comparing high vs. low bread intake were retained unless it was impossible to discern fermented food consumption (i.e., described only within broader categories such as cereals). Primary outcomes of interest were measures of glucose homeostasis or insulin, including both postprandial responses in short-term studies and fasting values in long-term studies, such as HbA1c, or other measures of insulin sensitivity. Therefore, studies without the collection of relevant blood samples for metabolic response analyses were excluded at the abstract screening stage. Incidence of T2D alone, however, was not a valid outcome for inclusion in full-text-level screening. Additionally, studies addressing diabetes incidence or risk were included provisionally at the abstract level, pending full-text evaluation for relevant glucose or insulin data. Studies assessing glucose changes solely during exercise were excluded. At the full-text level, studies assessing the GI of breads when a valid control was present were also included. Data on adverse effects for assessment of safety and effects on gut parameters, for possible mechanisms of action, were noted when available.

#### 2.1.3 Data extraction

A standardized data extraction form was used. Each study was independently reviewed by two researchers. Discrepancies were resolved by discussion. Extracted data included general information about the study, such as the study design, participant characteristics, and the study location. Additionally, details about the fermented breads and the controls used were extracted, along with the outcome measures and the detected effect. Depending on the type of control identified, studies were clustered into three specific research questions. Finally, adverse events and mechanistic hypotheses described in the studies were collected.

#### 2.1.4 Risk of bias (RoB) and quality assessment

For each included study with an adequate study design, the risk of bias was evaluated using the “Revised Cochrane risk-of-bias tool for randomized trials” (RoB 2) (23). The outcomes were visualized using Robvis (24). Potential sources of bias across multiple domains were considered, including the randomization process, period and carryover effects, adherence to interventions, missing outcome data, outcome measurement, and selection of reported results. Each domain was evaluated independently by two researchers, and an overall risk-of-bias judgment was subsequently assigned to each study, resolving discrepancies through discussion.

#### 2.1.5 Data synthesis

A descriptive synthesis was conducted. Due to differences in study designs and outcomes, as well as obtained results of risk of bias assessment, meta-analysis was not performed. The strength of the evidence was assessed from the identified studies with respect to each of our questions, pooling all considered glycaemia/insulinaemia outcomes together to identify any effect on glucose metabolism in general. Criteria that were applied consisted in the number of studies identified, their quality (considering the RoB assessment) and the consistency of the effects observed, in a shorten form of the GRADE approach (25). We aimed at evaluating the totality of evidence using the EFSA framework, as mentioned in section 2.3, using three grades (no or very limited, neither convincing nor sufficient, convincing and sufficient) and not using the GRADE system (with 4 grades) that focuses on clinic recommendations. The fact that we favor the EFSA evaluation justifies why a complete GRADE is not conducted. Still, for the evaluation of the evidence from the identified human studies, four levels of evidence were considered: (i) a high level (strong evidence), if more than 10 randomized control trials (RCT) were found, with the majority displaying a low-risk of bias evaluation and none a high risk and the vast majority reporting a consistent effect; (ii) a moderate level if at least 10 human studies (RCT or cohort studies) were available, with a maximum of one study with a high-risk of bias and the majority reporting a consistent effect; (iii) a low level of evidence if at least 10 human studies (RCT or cohort studies) were available, a minority displaying a high RoB and a majority reporting a consistent effect; (iii) a very low level of evidence if either only few human studies were found, and/or most identified studies showed a high RoB and/or no consistency in the reported effects were found.

### 2.2 Narrative framework for the systematic review

In addition to the systematic review, complementary narrative sections are included to provide context on bread characteristics, fermentation processes, and potential mechanisms of action. These sections were not part of the systematic evidence synthesis and are presented as complementary sections addressing important parts of an EFSA dossier. They did not influence the study selection or assessment of evidence from human studies (as described in §2.1.5) but were taken into account for the overall evidence assessment (as described below in §2.3).

#### 2.2.1 Characteristics of the fermented foods

Narrative synthesis was used to describe product characteristics, drawing on data from included studies and complementary sources (e.g., Codex Alimentarius and AACC standards). Attributes such as fermentation type, microbial strains, processing parameters, physical quality characteristics, proximate composition, and bioactive components that may affect glycaemic responses were also included.

#### 2.2.2 Supportive evidence: mechanism of action and bioavailability

Similarly to the product characterization, selected studies were screened for information on possible mechanisms of action and bioavailability of bioactive compounds. However, as this information was sparse, we complemented it with additional literature, particularly adding animal and *in vitro* studies. This strategy offered a deeper understanding of the involved mechanisms, including the potential role of the gut microbiota in mediating the effect of bread on glycaemia, as well as bioaccessibility and absorption of bioactive compounds from bread.

#### 2.2.3 Safety of fermented foods

All included studies were screened for adverse effects reported. This information was complemented with additional relevant literature.

### 2.3 Summary of the systematic and non-systematic parts of the review

We evaluated the current collective evidence using systematic and non-systematic parts of this review, taking into account the results for the human study search, the coherence/plausibility of the observed effects, and the characteristics of fermented breads. Therefore, three levels of overall evidence were defined, as used for an EFSA dossier: (i) a high level of evidence (“convincing and sufficient”) if strong evidence was found from the human studies, the mechanism of action pathways are plausible and bioactive compounds are well described; (ii) a low level of evidence (“neither convincing nor sufficient”) if there was either a strong evidence from human studies but lack of knowledge upon pathways and/or bioactives or a low evidence from human studies but well characterized bioactives and pathways; (iii) a very low level of evidence (“no or very limited”) if both the evidence from human studies was very low and the characterization of bioactives and pathways was limited.

## 3 Results and discussion

To understand how bread consumption could affect glycaemic regulation, we first summarize the characteristic of bread as a food type, then analyze the systematically selected human studies, report on possible mechanisms explaining the observed effects and finally conclude on the overall evidence.

### 3.1 Characterization of the food/constituent

#### 3.1.1 Food description

Bread is a staple food consumed daily by billions of people worldwide. In many societies, it serves as the primary source of carbohydrates due to its wide availability and affordability. Bread is a bakery product; the dough of bread is prepared primarily from flour of cereals and water, with the addition of one or more leavening agents (biological or chemical), and salt. Although the Codex Alimentarius does not provide an explicit definition of bread, it classifies bread and similar bakery products under the category *“bread and ordinary bakery wares”* (Category 07.1) within the General Standard for Food Additives (GSFA). This category specifies the permitted uses and restrictions of food additives, reflecting the importance of bread as a dietary staple. Similarly, the EU Food Categorization (Annex II of Reg. 1333/2008) classifies bread under Bakery wares—Bread and Rolls defining these products as food items prepared mainly from cereal flour or cereals that have undergone processing, such as baking, steaming, or extrusion.

Considering breads produced using biological leavening agents, two subtypes of bread can be distinguished: bread fermented by baker’s yeast and bread fermented using sourdough (11). Baker’s yeast-fermented bread is typically made with refined wheat flour, water, *Saccharomyces cerevisiae*, and salt. It is leavened with baker’s yeast and undergoes a relatively short fermentation. The widespread use of *S. cerevisiae* as the primary leavening agent in industrial bread production is driven by the need for consistency, rapid production and uniform texture and flavor.

Sourdough bread is leavened by fermentation with lactic acid bacteria (LAB) and yeasts, either spontaneously or using a starter culture. It undergoes a longer fermentation process than bread produced using baker’s yeast. This typically results in a sour flavor and modifies the nutritional profile through the production of a range of bioactive compounds that are absent in breads fermented exclusively with baker’s yeast (13). The microbiota involved in sourdough fermentation varies depending on the ingredients and fermentation conditions, but typically includes bacterial species such as *Fructilactobacillus sanfranciscensis*, *Limosilactobacillus fermentum*, *Lactiplantibacillus plantarum*, and *Levilactobacillus brevis*, along with yeasts such as *S. cerevisiae* and *Candida milleri* (26, 27). Recently, a starter culture containing the LAB *Companilactobacillus crustorum* LMG 23699 and the yeast *Wickerhamomyces anomalus* IMDO 010110 has been developed for sourdough fermentation (28).

Bread is produced primarily from wheat flour (either refined or whole grain) (29–35). Additionally, various other cereals and grains such as rye, barley, oats, millet, spelt, and rice are also incorporated in bread formulations (27, 31, 36–43). To improve the technological properties, sensory quality and nutritional profile of bread, additional ingredients may be introduced such as malt, ascorbic acid, gluten, flour from pulses, milk powder, flaxseed, semolina, *β*-glucan, starch, fat, olive oil, and sugar (33, 37, 44–46). The yeast-leavened bread may also be acidified by lactic and acetic acid to mimic the sensory properties of sourdough bread (31).

#### 3.1.2 Production methods

Both *S. cerevisiae* and sourdough-fermented breads involve similar processing steps, including mixing, kneading, proofing, and baking. However, sourdough bread production involves a longer fermentation as sourdough is a co-ferment, and the its microbiota requires a relatively long time to adapt (11).

In the industrial process of baker’s yeast-leavened bread, all ingredients like flour, water, *S. cerevisiae,* and salt are mixed to obtain dough prior to proofing. The dough is then kneaded for approximately 5–7 min, followed by a bulk proofing lasting 20–45 min at 28–38 °C and 80–85% relative humidity. After bulk fermentation, the dough is divided, shaped, and subjected to a final proofing stage for 30–60 min at 34–38 °C and similar humidity levels (31, 40, 45, 47). Baking is typically carried out at temperatures between 175–250 °C for 20–45 min, depending on the type and size of the bread (40, 45, 47, 48). To produce a glossy and well-developed crust, a short burst of steam is often applied at the beginning of baking. Some specialized methods use a first temperature holding step (e.g., at 70 °C for about 1 h), followed by baking at lower temperatures, such as 165 °C (35). Achieving consistent and standardized quality is generally easier in bread leavened with baker’s yeast due to its uniform microbial composition.

In sourdough bread production, the basic processing steps are similar. However, there is no standard formula or procedure for sourdough fermentation, leading to significant microbial variation within the sourdough culture. Factors such as flour type, water quality, and fermentation temperature contribute to variability in microbial growth, leavening performance, and the formation of bioactive compounds, all of which, in turn, influence the final product’s characteristics and, consequently, its potential health impact, sensory properties, and shelf life.

There are two broad types of sourdough ferment. Type 1 sourdough is the spontaneous fermentation of a mixture comprising flour and water at ambient temperature. In this type of sourdough ferment, the fermentation process requires daily “feedings” at a specific ratio of flour suspension, and it continues until the pH reaches approximately 4.0 ± 0.2. The production of type 1 sourdough generally requires 7–15 days (43, 49). Type 2 sourdough fermentation aims to expedite fermentation using specific LAB and yeast cultures. In type 2 fermentation, the desired pH level is achieved within 24–48 h without daily feeding (26, 28, 50).

After sourdough ferment preparation, it is incorporated into the final dough mixture along with the remaining bread ingredients, and the dough is kneaded. Afterward, the dough undergoes a two-step fermentation process. First, bulk fermentation is carried out for approximately 60–120 min at a temperature of 28–29 °C and 66–80% relative humidity, allowing for the initial development of gas retention and flavor compounds (33, 44). After this stage, the dough is divided and shaped, followed by a second proofing phase lasting 30–180 min at 30–38 °C and similar humidity levels (33, 43, 44). The proofing period can be reduced to 60 min by using Baker’s yeast in addition to sourdough (26). Finally, the fully proofed dough is baked at a temperature varying between 165 and 250 °C for 30–80 min, depending on loaf size, dough composition, and the desired crust and crumb characteristics (30–33, 35, 40, 42, 44, 45).

It should be noted that sourdough breadmaking practices vary substantially across studies and traditions, encompassing differences in flour type, water content, fermentation time and temperature, back-slopping regimes, and starter microbiota, so that the label “sourdough bread” covers a heterogeneous set of products (51). This heterogeneity is a further factor limiting the direct comparability of the intervention studies discussed below.

#### 3.1.3 Analytical methods

Processing and the type of ferment used can alter crumb structure, texture, composition, and bioavailability of different compounds in bread, which might affect the glycaemic responses to bread consumption (43).

The main physical characteristics of bread fermented with both sourdough and baker’s yeast include its specific volume, its texture and a homogeneous structure with small, homogeneous pores. Specific volume is measured by calculating the ratio of bread volume (mL) to weight (g) using the rapeseed displacement method AACC 10.05.01 (52). Porosity and internal microstructure can be examined via X-ray imaging, which assesses air cell distribution, the integrity of the protein matrix, and the extent of starch gelatinization (45). Texture profile analysis uses a texture analyzer to evaluate the hardness, springiness, cohesiveness, chewiness, and resilience of bread crumbs (53).

The physical and microstructural properties of bread play important roles in determining starch digestibility and influencing glycaemic response. In a study of rye crispbread specifically, Zamaratskaia et al. (43) reported that white bread exhibited greater starch gelatinization and amylose leakage compared to sourdough bread. Furthermore, in the same study the crispbreads were characterized with a more heterogeneous structure, with bran fragments dispersed throughout the matrix. These fragments were notably larger in unfermented samples than fermented ones. In addition, lamellae in the crumb of yeast-fermented wheat and unfermented rye crispbreads were thinner than those observed in sourdough-fermented rye crispbreads. These structural distinctions were proposed by Zamaratskaia et al. (43) to contribute to a slower disintegration rate of unfermented rye crispbreads, which can ultimately result in delayed glucose absorption and a reduced insulin response. Moreover, increased bread crumb firmness was associated with limited starch digestibility, potentially due to enhanced agglomeration and crystallinity of starch fractions. The development of compact amylopectin–amylose networks may further reduce starch’s susceptibility to amylolytic enzymes and hinder enzymatic penetration (54). Additionally, specific microstructural features such as a dense matrix and an amylose layer surrounding starch granules are thought to play a key role in reducing starch accessibility and digestibility (43). These findings are however related to rye crispbread and may not apply to conventional bread, whose crumb structure and starch gelatinization differ substantially.

The chemical composition of bread is determined using standardized analytical methods. Total protein content is quantified by the Kjeldahl method (Method no: 46.12.01), total fat content is measured using ether extraction (Method no: 30.10.01), and total ash content is determined by incinerating the sample at 575–590 °C until a constant weight is obtained (Method no: 08.01.01) 01 (52). Starch (Method no: 76.13.01), dietary fiber (Method no: 32.07.01), resistant starch and *β*-glucan content (Method no: 32.22.01) are highly correlated with the glycaemic response of bread, and they are assessed using the enzymatic hydrolysis method 01 (52).

Fermentation has been shown to increase the content of bioactive compounds, such as phenolics, which may help lower glycaemic and insulin responses (55). The interactions between starch and components such as lipids, organic acids, or polyphenols reduce starch digestibility, as these compounds limit the accessibility of amylolytic enzymes to amylose/amylopectin or inhibit amylase activity (56). Therefore, quantifying these components in bread is important. In sourdough bread, fermentation lowers pH and increases the concentration of organic acids, mainly lactic and acetic acids. Therefore, parameters such as pH, total titratable acidity, and lactic and acetic acids are routinely monitored in sourdough bread. The pH value is measured by a pH meter, and the titratable acidity is determined by titrating a homogenized sample with 0.1 N NaOH (Method no: 02.31.01) (52). Organic acids are quantified by High-Performance Liquid Chromatography (HPLC) (27).

The acidity generated during sourdough fermentation is among the factors that may contribute to a lower glycaemic response of sourdough bread compared with matched baker’s-yeast breads in some studies. Organic acids present in the dough have been shown *in vitro* to reduce the rate of starch hydrolysis by promoting interactions between starch and gluten during starch gelatinization. Moreover, they may support the debranching of amylopectin moieties during baking, and debranched amylopectin may lead to a high level of resistant starch, which, in turn, can act as a physical barrier against enzymatic attack by *α*-amylase (48, 57). Moreover, the organic acids generated in sourdough fermentation could affect satiety by delaying gastric emptying (43, 58). It should be emphasized, however, that these mechanistic observations do not translate into a consistent low glycaemic index for sourdough bread across studies as presented below.

A further characteristic of bread that should be analyzed to understand its impact on glycaemia is the solubility of its constituents. Sourdough fermentation, besides increasing resistant starch content as mentioned above, also increases solubility of dietary fiber and protein, and decreased molecular weight of *β*-glucans in bread (43, 59, 60). Higher solubility and lower molecular weight of dietary fibers may increase their fermentability in the colon, thereby contributing to increased satiety (43). On the other hand, the high molecular weight of soluble fiber contributes to increased digesta viscosity, which might reduce gastric emptying rate and the digestion and absorption rate of nutrients (30, 43).

Therefore, detailed product characterization should be presented in future studies. This includes raw material specifications, such as the types of raw ingredients and the fermentation process (e.g., yeast/bacteria type and fermentation time), which can be translated into well-defined bread formulations and preparations. For sourdough bread, the microbial composition of the sourdough/dough should be given, along with physicochemical parameters (moisture, pH, organic acids). For both kinds of bread (sourdough and yeast) structural characteristics, such as internal microstructure and texture profile, and particularly chemical characteristics, such as details on starch and fibers content (including resistant starches), as well as their degree of gelatinization, would facilitate comparisons between studies and deepen our understanding of possible mechanisms of action. For this purpose, we recommend using multiple, validated, and reproducible analytical methods to support effective comparisons.

### 3.2 Identification of pertinent human efficacy studies

#### 3.2.1 Overview of identified studies

To answer the three specific research questions about the effect of bread consumption on glycaemia, studies from the literature based on pre-defined PICO criteria were selected. Checking them first, only for PIO criteria allowed us to identify 65 studies, from which only 27 were included. The main reason for exclusion at this step was that the effect of bread was compared with that of a glucose solution. As we wanted to identify the effect of fermented bread as a whole and not the effect of fiber specifically, studies directly comparing two breads only differing in their fiber content were not included, except for long-term studies, in which a change in glycaemia for each bread separately between start and end of intervention was considered.

A criterion for the study design was not defined, as different study designs could help answer our questions. However, the included studies were exclusively intervention studies. The main reasons for the absence of observational studies were that bread was often pooled in one category with other cereal products, mostly separated into refined and wholegrain, precluding the analysis of the effect of bread alone, and that the outcome assessed was typically the prevalence of T2D without a direct measurement of blood parameters of glycaemia and insulinaemia.

##### 3.2.1.1 Population

In line with EFSA guidelines, we included studies with healthy participants. However, the population characteristics still varied across the studies, as we defined “healthy” as not taking medication (with specific exceptions) and as the absence of a diagnosed disease. However, we included studies in participants with a high BMI or those with pre-diabetes. Among the 27 selected studies, 23 comprised healthy participants, two involved participants with pre-diabetes/impaired glucose tolerance, and two included both healthy and prediabetic participants (one separately, one as a mixed population).

We found 21 studies from Europe (eleven from Nordic countries), three from North America (Canada), one from the Middle East, one from China, and one from New Zealand, revealing an uneven distribution of data across continents that is not directly related to the importance of bread in local nutrition. The implications of this distribution for the generalization of our findings are discussed in Section 3.5.

The mean study population size considered was rather small (*n* = 18.1), ranging from 6 to 64 participants. Both sexes were represented, either in mixed populations (24 studies) or in studies with only male participants (33, 45) and a unique study focusing on postmenopausal women (61). This led to a total of 488 participants, among them at least 237 females and 190 males (the exact sex distribution in several studies was not reported).

We considered only adult populations; therefore, all included studies had participants aged 18 or older. Still, the mean age and age range for participants varied across studies (from 22.1 ± 3.6 to 60.1 ± 12.1 y).

##### 3.2.1.2 Outcomes

Most studies (*n* = 24) assessed both glucose and insulin or C-peptide, some calculated additional parameters such as insulin resistance or sensitivity, and a few measured other glycaemia-related parameters (such as fructosamine, glucose-dependent insulinotropic polypeptide (GIP) or glucagon- like peptide-1 (GLP-1)). Most studies reported postprandial effects on glycaemic parameters after either the intake of a piece of test bread only or a standardized meal containing the test bread (*n* = 20), while one study reported on similar responses after a standard breakfast following an evening meal with the test bread (62). Only seven studies reported on long-term effects of daily bread consumption, replacing usual bread and/or cereal products with the test bread within the usual diet.

##### 3.2.1.3 Intervention

All selected studies were intervention studies in which participants consumed different types of bread during a single meal (post-prandial focus) or over several days (1 to 12 weeks). Most studies used a crossover design.

We could assess different aspects of the effect of fermented bread on glycaemia depending on the control used in the studies. We identified 12 studies that compared yeast-fermented versus sourdough breads with the same cereal/flour quality, allowing the effect of fermentation type to be evaluated (Table 1). Ten studies provide insights into the general effects of fermentation, comparing fermented bread with unfermented cereal products such as unfermented bread, pasta, or porridge (Table 2). Finally, we identified six studies that used long-term interventions, allowing a comparison of glycaemic parameters before and after consumption of the tested breads without a control (Table 3).

**Table 1:** Overview of the studies meeting our inclusion criteria for the comparison of yeast and sourdough fermented breads.

First author, year of publication (ref)	Global region	Study design, focus	Population		Glycaemia related outcomes
			Health status (*n*)	Mean body weight	
Pagliai, 2020 (27)	Europe	RCT, CO; 4 weeks intervention	Healthy participants (17)	Normal weight	Change in fasting blood glucose
Chatonidi, 2025 (28)	Europe	RCT, CO; postprandial focus	Healthy participants (44)	Normal weight	Serum C-peptide and plasma glucose responses (at different time points, curve (graphical data) + AUC over 240 min. After consumption (tabular data))
Dall’Asta, 2022 (44)	Europe	RCT, CO; postprandial focus	Healthy participants (12)	Normal weight	Capillary blood glucose and plasma insulin responses (whole curve and iAUC (graphical data) up to 120 min., peak (tabular data))
Najjar, 2009 (33)	North America	RCT, CO; postprandial focus (incl. second meal)	Healthy participants (10)	Elevated BMI	Blood glucose and serum insulin as well as GIP and GLP-1 responses (whole curve (graphical data) up to 5 h after first meal, AUCs (tabular data) for 5 h and 3 h after first meal and 2 h after second meal; Insulin sensitivity index (calculated)
Bo, 2017 (30)	Europe	RCT, CO; postprandial focus	Healthy participants (16)	Normal weight	glucose and insulin responses (different time points, curve and AUCs up to 120 min after meal (graphical and tabular data))
Novotni, 2012 (26)	Europe	RCT, CO; postprandial focus	Healthy participants (11)	Normal weight	Finger prick capillary blood glucose response up to 120 min after meal (only curve as graphical data) + glycaemic index
Borczak, 2011 (47)	Europe	RCT, CO; postprandial focus	Healthy participants (15)	Normal weight	Glucose response up to 120 min after meal (curve (graphical data) and whole iAUC (tabular data)) + glycaemic index
Fredensborg, 2010 (49)	Oceania	RCT, CO; postprandial focus	Healthy participants (10)	Normal weight	Plasma glucose response up to 120 min. (graphical data) + glycaemic index
Darzi, 2012 (63)	Europe	RCT, CO; postprandial focus	Healthy participants (20)	Normal weight	Plasma insulin and glucose responses up to 180 min after meal (curves: graphical data), Insulin sensitivity
Liljeberg, 1995 (48)	Europe	RCT, CO; postprandial focus	Healthy participants (11)	Normal weight	Finger prick capillary blood glucose (up to 180 min.) and insulin responses (up to 120 min.) (curves: graphical data + different AUCs and time points (tabular data))
Scazzina, 2009 (65)	Europe	RCT, CO; postprandial focus	Healthy participants (8)	Normal weight	Finger prick capillary blood glucose response up to 120 min. (whole curve and AUC as graphical data)
Maioli, 2008 (64)	Europe	RCT, CO; postprandial focus	Prediabetic (16)	Elevated BMI	Plasma glucose and insulin responses (different AUCs, up to 180 min. as graphical data (curves))

Ref nb, reference number; RCT, randomized control trial; CO, cross-over design; (i)AUC: (incremental) area under the curve. Mean body weight is classified as “normal weight” when the mean BMI of the population is ≤25 kg/m^2^ and as “elevated weight” when the mean BMI is >25 kg/m^2^. The “Glycaemia related outcomes” column specifies the time window of the response measurement and whether the data were available in graphical or tabular form, as these aspects underlie the decision not to perform a meta-analysis on this body of evidence.

**Table 2:** Overview of the studies meeting our inclusion criteria for the comparison of fermented breads with non-fermented cereal-based controls (unfermented breads, pasta, porridge, or boiled kernels).

First author, year of publication (ref)	Global region	Study design, focus	Population		Glycaemia related outcomes
			Health status (*n*)	Mean body weight	
Johansson, 2015 (66)	Europe	RCT, CO; postprandial focus. Comparator: unfermented whole-grain rye crispbread (yeast-fermented vs. unfermented, same flour origin and composition)	Healthy participants (23)	Normal weight	Plasma glucose and serum insulin responses up to 230 min after a standardized breakfast (curves: graphical data only; AUC 0–120 min and 0–230 min reported in text)
Zamaratskaia, 2017 (43)	Europe	RCT, CO; postprandial focus. Comparator: unfermented whole-grain rye crispbread (sourdough-fermented vs. unfermented)	Healthy participants (24)	Normal weight	Plasma glucose and insulin responses up to 230 min (curves: graphical data only; insulin AUC 0–125 min and 0–230 min reported in text)
Eelderink, 2015 (45)	Europe	RCT, CO; postprandial focus. Comparator: unfermented wheat flat bread (yeast-fermented vs. unfermented; same study also includes a pasta arm)	Healthy participants (10)	Normal weight	Plasma glucose and insulin responses up to 360 min after meal (different time points, peak values, iAUC 0–120 min and 0–360 min; partly graphical, partly tabular data)
Al Dhaheri, 2017 (29)	Middle East	RCT, CO; postprandial focus. Comparator: unfermented regag bread (yeast-fermented Arabic bread vs. unfermented regag)	Healthy participants (25)	Normal weight	Glycaemic index and glycaemic load (tabular data only; no inferential statistics reported)
Kristensen, 2010 (67)	Europe	RCT, CO; postprandial focus. Comparator: pasta (refined-wheat bread vs. refined-wheat pasta; whole-grain wheat bread vs. whole-grain wheat pasta)	Healthy participants (16)	No BMI reported (mean age 24 y; healthy young adults)	Blood glucose response over 180 min (different time points and AUC; partly graphical, partly tabular data)
Liljeberg, 1999 (37)	Europe	RCT, CO; postprandial focus, including second-meal effect. Comparator: pasta (white-wheat bread vs. durum-wheat spaghetti)	Healthy participants (10)	No BMI reported (age range 22–57 y)	Glycaemic and insulinaemic indices; second-meal glucose response (45–70 min) and insulin response (0–45 min) (partly graphical, partly tabular data)
Nilsson, 2008 (62)	Europe	RCT, CO; postprandial focus, with response measured at a standard breakfast on the morning after a test evening meal. Comparator: pasta (white-wheat bread + barley DF vs. spaghetti + barley DF)	Healthy participants (20)	Normal weight	Fasting and postprandial blood glucose and serum insulin responses to a standard breakfast the morning after the test evening meal (curves and AUC; graphical and tabular data)
Liljeberg, 1996 (38)	Europe	RCT, CO; postprandial focus. Comparator: porridge (high-fiber barley bread vs. high-fiber barley porridge)	Healthy participants (9)	Normal weight	Capillary blood glucose and insulin responses; glycaemic and insulinaemic indices calculated (different time points and AUCs; partly graphical, partly tabular data)
Rosén, 2009 (41)	Europe	RCT, CO; postprandial focus. Comparator: porridge (white-wheat bread vs. porridge; endosperm rye bread vs. porridge; whole-grain rye bread vs. porridge—three matched comparisons within the same study)	Healthy participants (12)	Normal weight	Capillary blood glucose and serum insulin responses (including AUC 0–30 min); glycaemic and insulinaemic indices (partly graphical, partly tabular data)
Rosén, 2011 (40)	Europe	RCT, CO; postprandial focus. Comparator: boiled rye kernels (whole-grain rye bread vs. whole-grain rye kernels)	Healthy participants (10)	Normal weight	Blood glucose and serum insulin responses; glycaemic profile, glycaemic index and insulinaemic index (different time points and AUCs incl. 120–270 min; partly graphical, partly tabular data)

Ref nb, reference number; RCT, randomized controlled trial; CO, cross-over design; (i)AUC, (incremental) area under the curve; DF, dietary fiber. Mean body weight is classified as “normal weight” when the mean BMI of the population is ≤25 kg/m^2^ and as “elevated weight” when the mean BMI is >25 kg/m^2^, in line with Table 1. Where mean BMI was not reported, this is indicated together with the available descriptors. The “Glycaemia related outcomes” column specifies the time window of the response measurement and whether the data were available in graphical or tabular form, as these aspects underlie the decision not to perform a meta-analysis on this body of evidence.

**Table 3:** Overview of the studies meeting our inclusion criteria for the general effect of fermented bread consumption on glycaemia.

First author, year of publication (ref)	Global region	Study design, focus	Population		Glycaemia related outcomes
			Health status (*n*)	Mean body weight	
MacKay 2012 (46)	North America	RCT, CO; 6 weeks intervention. Comparison: refined white wheat bread vs. whole-grain wheat sourdough bread; before-after within each subgroup	Normoglycaemic/Normoinsulinaemic adults (*n* = 14) and hyperglycaemic/hyperinsulinaemic adults (*n* = 14)	elevated weight (both subgroups; mean BMI 26.5 kg/m^2^ in NGI and 35.7 kg/m^2^ in HGI)	Fasting plasma glucose, insulin and HOMA-IR; oral glucose tolerance test (OGTT) glucose and insulin responses; insulinogenic index (calculated). Day 1 vs. day 43 within each bread arm (tabular data for fasting parameters; OGTT responses presented graphically). No formal within-group day 1 vs. day 43 statistical comparison reported.
Pagliai 2020 (27)	Europe	RCT, CO; 4 weeks intervention. Comparison: ancient-grain sourdough bread vs. ancient-grain baker’s-yeast bread; before-after within each arm	Clinically healthy participants (*n* = 17)	Normal weight	Change in fasting blood glucose between baseline and end of each 4 week bread period (tabular data only).
Lappi 2014 (68)	Europe	RCT, CO; 4 weeks intervention per test bread, preceded by a 4 week white-wheat run-in. Comparison: end-of-run-in vs. end-of-test-bread period for whole-grain sourdough rye bread and for bioprocessed rye-bran + wheat bread	Healthy adults reporting gastrointestinal symptoms after grain ingestion (*n* = 21)	No mean BMI reported (range 19–30 kg/m^2^)	Fasting plasma glucose and insulin; postprandial glucose and insulin responses to a test meal up to 120 min; calculated first-phase insulin secretion and disposition index. End-of-run-in vs. end-of-each-test-bread period (tabular data for fasting values and calculated indices; postprandial responses partly graphical, partly tabular).
Todesco 1991 (35)	North America	RCT, CO; 1 week intervention. Comparison: white wheat bread vs. propionate-enriched white wheat bread; before-after within each arm	Healthy adults of ideal body weight (*n* = 6)	No BMI reported (described as ideal body weight; mean weight 60.5 ± 12.8 kg)	Postprandial blood glucose response to a standard meal (AUC). Start vs. end of each 1 week bread period (tabular data only).
Ren 2018 (39)	Asia	Open-label, self-controlled clinical trial; 12 weeks intervention with foxtail-millet steamed bread; before-after comparison	Free-living adults with impaired glucose tolerance (*n* = 64)	Elevated weight (mean BMI 26.0 kg/m^2^)	Fasting plasma glucose, insulin, C-peptide, fructosamine and GLP-1; 2 h glucose and insulin responses to an OGTT; HOMA-IR and HOMA-IS (calculated). Baseline vs. week 12 (tabular data only).
Juntunen 2003 (61)	Europe	RCT, CO; 8 weeks intervention. Comparison: high-fiber rye bread vs. white wheat bread; before-after within each arm	Healthy postmenopausal women, including 3 with impaired glucose tolerance (*n* = 20)	No mean BMI reported (range 20–33 kg/m^2^)	Fasting plasma glucose and insulin; glucose and insulin responses to a frequently sampled intravenous glucose tolerance test (FSIGTT); calculated insulin sensitivity, glucose effectiveness and acute insulin response (AIR). Baseline vs. end of each 8-week bread period (tabular data only).

Ref nb, reference number; RCT, randomized controlled trial; CO, cross-over design; (i)AUC, (incremental) area under the curve; OGTT, oral glucose tolerance test; FSIGTT, frequently sampled intravenous glucose tolerance test; HOMA-IR, homeostatic model assessment of insulin resistance; HOMA-IS, homeostatic model assessment of insulin sensitivity; NGI, normoglycaemic/normoinsulinaemic; HGI, hyperglycaemic/hyperinsulinaemic. Mean body weight is classified as “normal weight” when the mean BMI of the population is ≤25 kg/m^2^ and as “elevated weight” when the mean BMI is >25 kg/m^2^, in line with Table 1. Where mean BMI was not reported, this is indicated together with the available descriptors. The “Glycaemia related outcomes” column specifies the time window of the response measurement and whether the data were available in graphical or tabular form, as these aspects underlie the decision not to perform a meta-analysis on this body of evidence.

### 3.3 Impact of the type of fermentation on the glycaemic effects of bread

Of the twelve studies comparing a sourdough with a similar yeast bread (Table 1, Supplementary file 3), only one intervention did not consider an acute, postprandial response, instead testing the effect of 4 weeks of daily consumption of the breads (27). All other studies assessed the postprandial responses after consuming the test breads, either alone (with only a glass of water) or as a breakfast (including same toppings/drinks between groups) (48, 63, 64). Test bread portions were mainly matched for carbohydrate content (50 g), whereas in three cases test breads were matched on weight per portion (100 g for Maioli et al. (64), 126 g for Darzi et al. (63) and 150 g for Chatonidi et al. (28)). Most studies considered breads made from wheat flour only (*n* = 8), including whole grain flour, white flour or both. One study assessed the effect of gluten-free breads based on rice, corn and buckwheat (26), one study used mostly barley with 20% wheat (48) and another wheat semolina and 30% corn (64) to produce their breads. In one study the information on the type of flour (white or whole meal) was missing (27). Considering the characterization of the breads, all included studies gave some information on the chemical composition of their bread, mostly nutritional composition or, for six studies pH or organic acids (27, 28, 33, 48, 63, 64). Only Pagliai et al. (27) reported on microbiological analyses of their sourdough. Scazzina et al. (65) and Liljeberg et al. (48) both analyzed the *in vitro* digestibility of starches from their breads, whereas Maioli et al. (64) reported on microstructural analyses using electron microscopy and Novotni et al. (26) on several physical parameters (specific volume, crumb firmness, viscosity).

All twelve studies measured a glycaemia parameter, three also calculated the GI of tested breads (26, 47, 49) and seven measured an insulinaemia parameter, either C-peptide (28) or insulin (30, 33, 44, 48, 63, 64). Pagliai et al. (27) reported that the change in fasting blood glucose (FBG) after a 4 week-intervention was not significatively different between the two groups, even if FBG increased after consumption of the yeast bread while no significant changes on FBG were observed with the sourdough treatment. Among the postprandial response studies that calculated the GI of breads, differences were observed depending on fermentation method in two studies. An incremental effect was observed by Novotni et al. (26), who tested different quantities of sourdough added to a standard yeast-based recipe and, found a significantly lower GI with 15% and 22.5% of sourdough, when compared to a yeast-fermented bread with no sourdough. Similarly, Borczak et al. (47) found a lower GI for their sourdough bread than the yeast control. In contrast, Fredensborg et al. (49) did not identify significant differences between breads prepared using different leavening agents (backer yeast, desem, sourdough).

Differences in other measures of the glucose response based on bread fermentation method were also reported with only Darzi et al. (63) noting no significant difference between control yeast and sourdough bread. All of the other studies found either a significantly lower overall response for sourdough vs. yeast bread (33, 44, 47, 65), or at least at some specific time points (26, 28, 30) or for partial Area Under the Curve (AUC) (48, 64).

From the seven studies reporting on insulinemia response, only Najjar et al. (33) and Darzi et al. (63) did not find any significant difference between breads using sourdough and yeast. The other five studies reported significantly lower insulinemia responses after sourdough than yeast breads, at least for some specific time points during the postprandial response (28, 30, 44, 48, 64).

In this specific question about the effect of leavening type, only Najjar et al. (33) reported on incretin release, measuring both GIP and GLP-1, which stimulate insulin production. Interestingly, even if in their study no significant differences in insulin release or calculated insulin sensitivity were found, the overall response of GLP-1 was significantly lower after sourdough than after yeast bread consumption and the second meal GIP response was also reduced after sourdough compared to after yeast bread.

In total 10 of the 12 studies, or five from seven, reported some positive effect on glycaemia, or insulin responses respectively, for sourdough compared to yeast bread consumption. A further consideration when interpreting comparisons between sourdough and baker’s-yeast breads is that the two production processes typically differ not only in the microbiota involved but also in the length of fermentation, which is generally longer for sourdough. Any effect observed in a sourdough vs. baker’s-yeast comparison may therefore reflect not only the different microbial activity but also the longer action of endogenous cereal enzymes (e.g., amylases, phytases and proteases) on the starch, phytate and gluten matrix. Very few of the included studies attempted to match fermentation times between the two arms or explicitly reported them, limiting the ability to disentangle the effect of fermentation type per se from that of fermentation duration.

#### 3.3.1 General impact of fermentation on the glycaemic effects of bread

From the 10 studies comparing fermented bread to a non-fermented control, four used a non-fermented flat bread (29, 43, 45, 66), four used pastas (37, 45, 62, 67), two porridge (38, 41), and one boiled kernel (40) as control products (Table 2, Supplementary file 4). The fermented breads were all produced using yeast, except for one study in which a sourdough bread was compared with an unfermented bread (43). Cereals used for food item preparation varied across studies, with wheat (29, 37, 41, 45, 62, 67) and rye (40, 41, 43, 66) being the most common, and only one study using barley (38). Similarly, the fiber content of the test products differed between studies, some comparing food items with white flour without fiber (29, 37), with the addition of dietary fiber (45, 62), with whole-grain flour (38, 40, 43, 66), or with both (41, 67). We only considered comparisons between food items of similar composition (cereal type and fiber content). All studies reported on the nutritional composition of the breads, with more or less details on fiber and starches, and for Zamaratskaia et al. (43), on organic acids. Only Rosén et al. (40, 41) performed an *in vitro* starch hydrolysis in both their studies. Only Eelderink et al. (45) analyzed the microstructure of their breads (density, porosity).

All studies assessed the postprandial response after a breakfast containing the test products, and, in the case of Liljeberg et al. (37), also after a standard second meal, with the exception of Nilsson et al. (62), who assessed the response after a standard breakfast following an evening meal made of the test products. Test breads were consumed as part of breakfast, mainly with cheese, in six studies (37, 38, 43, 45, 66, 67); in the four other studies, they were only accompanied by a glass of water. Most studies adjusted the amount of tested food to a defined amount of available starch (30–50 g) (29, 37, 38, 40, 41, 45, 62, 67). All 10 studies measured postprandial glucose response; five calculated the GI of the tested breads (29, 37, 38, 40, 41); and eight also measured the insulin response.

The four studies comparing fermented and unfermented bread yielded variable results. While two studies did not report any significant difference for the glucose response despite a lower insulin response (AUC) after consumption of the unfermented crisp breads compared to either yeast (66) or sourdough fermented breads (43), Eelderink et al. (45) found a lower insulin response (AUC, different time points, peak value) and a lower glucose peak value after unfermented bread consumption. In contrast, fermented Arabic bread was found to have a lower GI than unfermented regag bread (29).

Of the four studies comparing fermented bread to pasta, three reported significantly higher postprandial responses after bread consumption: either in glucose peak value (45), total AUC or time-point values for both refined and whole grains (67), or GI and second-meal responses (37). Similarly, two of these studies, which also assessed insulin, showed significantly lower insulin responses after pasta than after bread consumption either as whole AUC (45) or as insulin index at different time points after the second standard meal (37). In contrast, Nilsson et al. (62) did not find a significant difference in either glucose or insulin response to a standard breakfast following an evening meal containing the test foods. The time between the evening meal and the test breakfast in the study by Nilsson et al. (62) was, however, longer than the time between the test breakfast and the standard midday meal in the study by Liljeberg et al. (38), which might explain the different results observed. Generally, it seems that pasta is more favorable than fermented breads for acute glycaemic responses, which is probably due to their high content of resistant starches.

We identified two studies that met our criteria and compared fermented breads to porridges. Both showed a significantly lower insulin response for the breads in the early phase (AUC 0–30 min), compared to the porridges, with a lower peak value for endosperm rye bread (41) and a lower insulin index for high-fiber barley bread (38). Whereas the insulin changes were not accompanied by a significant difference in glucose response for Liljeberg et al. (38), Rosén et al. (41) found lower early response (AUC 0–30 min) of glucose for their rye breads compared to the rye porridges.

Finally, Rosén et al. (40) compared a fermented bread to boiled kernels (both prepared from whole grain rye), and did not detect any significant difference in the glucose response (GI, peak glucose, and AUC) but found a significantly higher late insulin response (AUC 120–170 min) for the kernels.

To summarize, the effect of fermented bread compared to unfermented cereal products on glycaemia is not generalizable. Few studies have compared fermented with unfermented breads, among them crispbreads with a markedly different structure from that of conventional breads, limiting direct assessment of a generic fermentation effect and highlighting the importance of structural characterization of breads. Where suitable comparators were described, the effects on glycaemia were conflicting and inconsistent, perhaps due to specific characteristics of the selected test products. Whereas, most unfermented breads and pasta produced lower glucose responses, particularly on insulin responses, porridges and boiled kernels could be less favorable than fermented breads. Still, for each case, the number of studies is too low to draw a clear conclusion.

#### 3.3.2 The effect of fermented bread consumption on glycaemia

Finally, we analyzed the overall effect of fermented bread consumption, instead of focusing solely on comparisons with unfermented cereal foods or between different fermentation types.

As no observational study comparing low versus high bread consumption was identified, we applied our other selection criteria and considered studies for which data were available before and after a longer intervention (Table 3, Supplementary file 5). Even if effects observed in such before-and-after comparisons without a control group eating no bread or a different quantity are to be interpreted carefully, we chose to comprehensively review all relevant evidence. From the six longer intervention studies identified, most tested at least one wheat-based bread (27, 35, 46, 61, 68), two considered high fiber/whole grain rye bread (61, 68), and one a foxtail millet bread (39). The nutritional composition of the bread was reported in only three studies (46, 61, 68). Organic acids and microbial composition were analyzed only in Pagliai et al. (27), whereas Todesco et al. (35) reported upon *in vitro* starch digestion analyses of their breads and Ren et al. (39) referred to previous work for similar analyses.

The period during which participants had to consume the tested bread daily ranged from 1 week (35) to 12 weeks (39). The daily dose of bread was either the same for each participant (from 90 g in Ren et al. (39), to an amount of bread corresponding to 150 g carbohydrate in Todesco et al. (35)), or was adapted to local nutrition guidelines (46), or defined as a minimum number of portions (61). In all studies, participants were advised to maintain their habitual diet and lifestyle, replacing usual breads and cereals with the test breads. All studies compared at least two different breads for their effect on fasting blood glucose, some also considered fasting insulin (39, 46, 61, 68) and few also analyzed the acute response to an oral glucose tolerance test (OGTT) (39, 46), a test meal (68) or a frequently sampled intravenous-glucose-tolerance test (FSIGTT) (61) before and after the intervention.

In most cases, the fermented bread interventions did not lead to a significant change in glycaemic outcomes. However, in one case, a negative effect was observed: Pagliai et al. (27) detected an increase in FBG after 4 weeks of the control wheat bread, whereas their sourdough bread did not induce a significant change. In few cases a positive effect was found on some outcomes: Lappi et al. (68) observed a decrease in the late insulin response (at 120 min, after a test meal) and an increase in the calculated disposition index after 4 weeks of wholegrain sourdough rye bread; Todesco et al. (35) detected a lower glucose response after a standard meal after 1 week of a propionate-enriched white wheat bread; Ren et al. (39) found, after 12 weeks of a foxtail millet bread, a high impact in subject with IGT with a decrease in FBG and 2 h-glucose response to an OGTT as well as a decrease in calculated insulin resistance index (HOMA-IR).

To summarize, except for a few specific breads and specific outcomes, bread consumption over several days or weeks was not associated with significant changes on glycaemia parameters in healthy participants. However, it must be noted that for all these studies, the observed effects were not controlled for, as we considered only changes within each group. Moreover, participants had consumed bread before joining the respective studies and were only advised to replace it with the test breads. None of the identified studies provided details on bread consumption before intervention, which limits the interpretation of the observed effects. This is probably because all studies, except Ren et al. (39), compared different types of bread. Observational studies comparing people eating variable quantities of bread would have provided stronger evidence for this research question, but no suitable studies were identified, due to the strict criteria of a healthy population and glycaemia outcomes as blood parameters.

#### 3.3.3 Risk of bias (RoB) assessment

**Figure 2:**
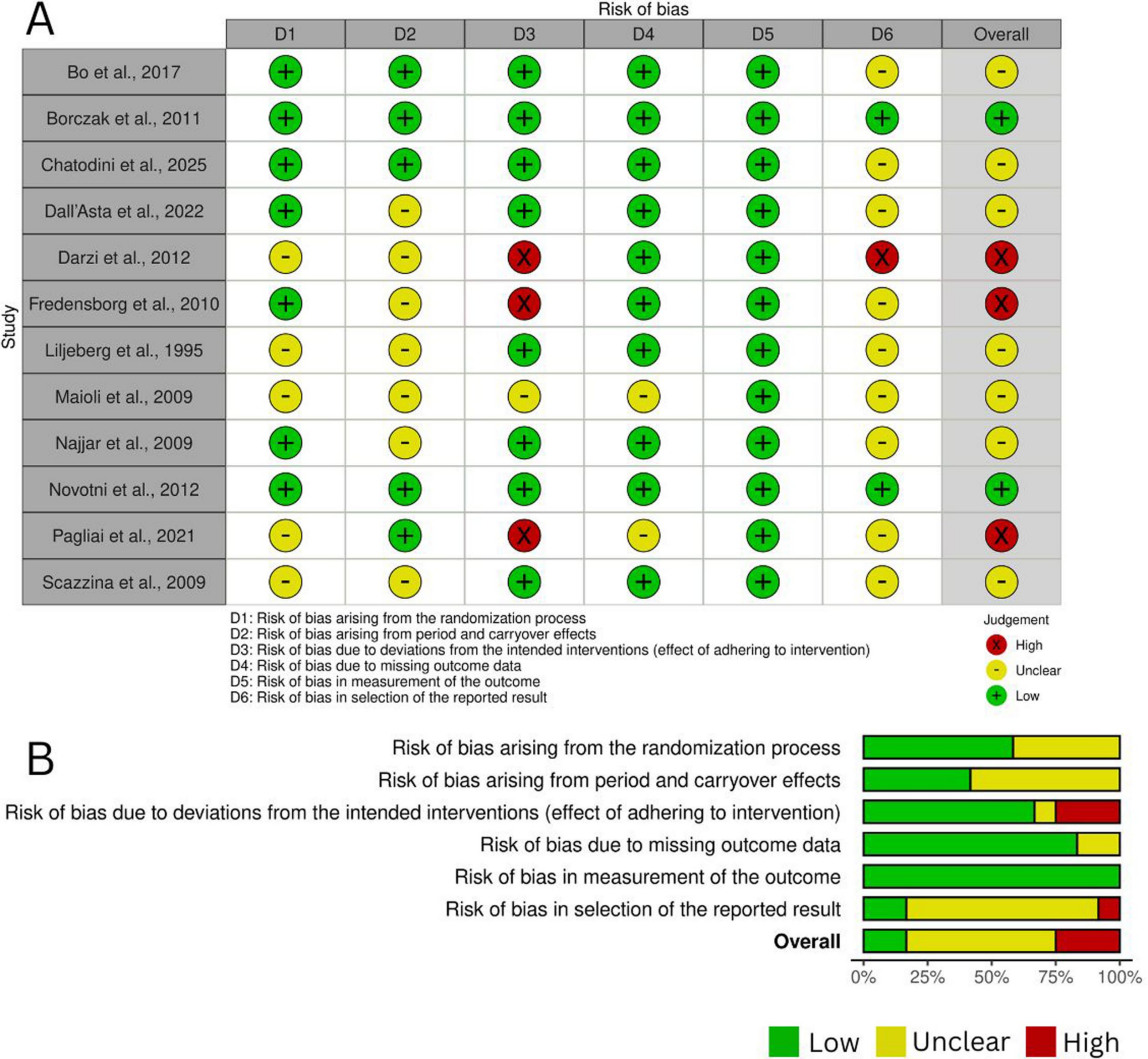
Risk of bias analysis of the studies assessing the impact of the type of fermentation on the glycaemic effects of bread. **(A)** Results for each study. **(B)** Summary per domain of risk.

**Figure 3:**
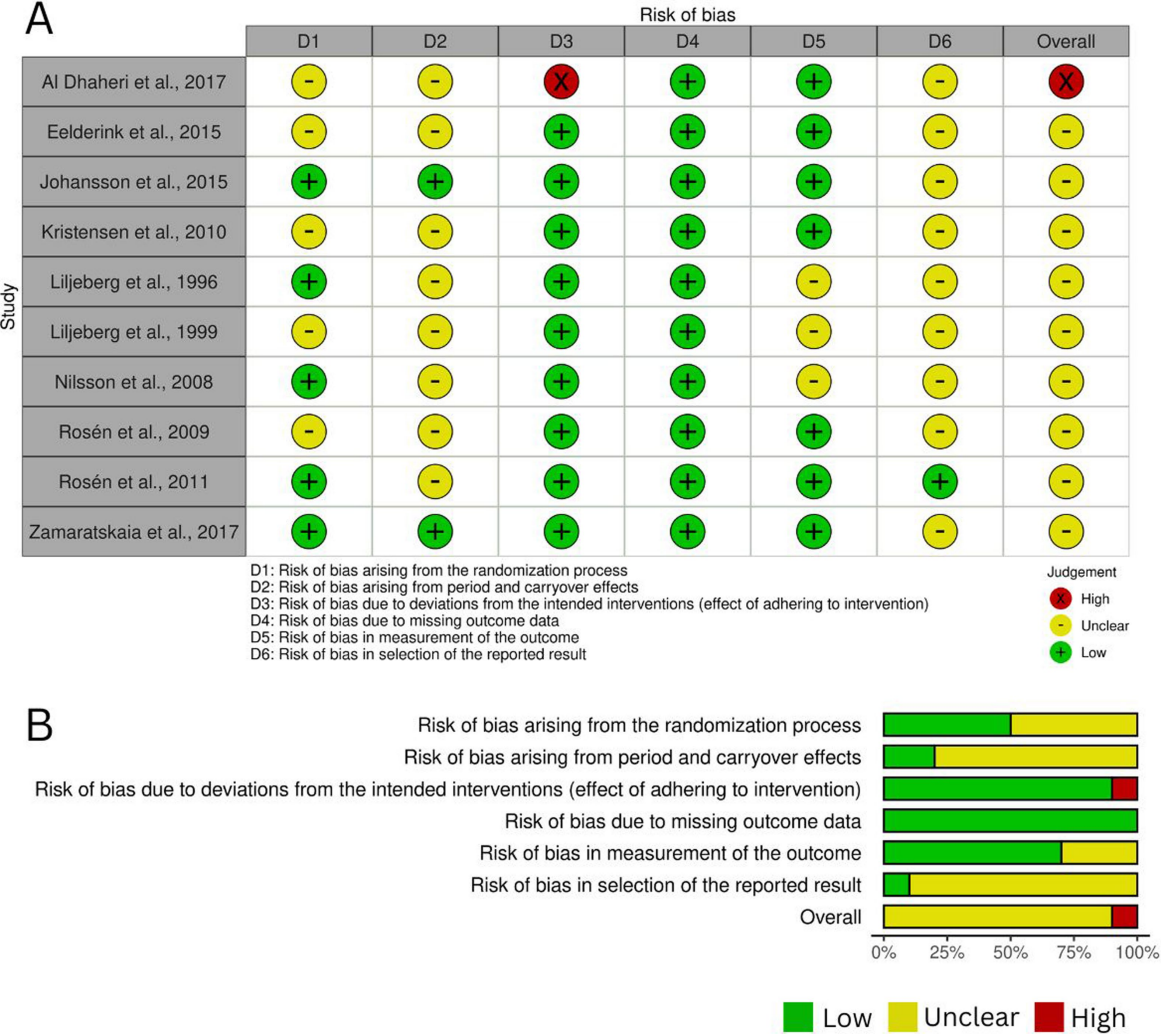
Risk of bias analysis of the studies assessing the general impact of fermentation on the glycaemic effects of bread. **(A)** Results for each study. **(B)** Summary per domain of risk.

**Figure 4:**
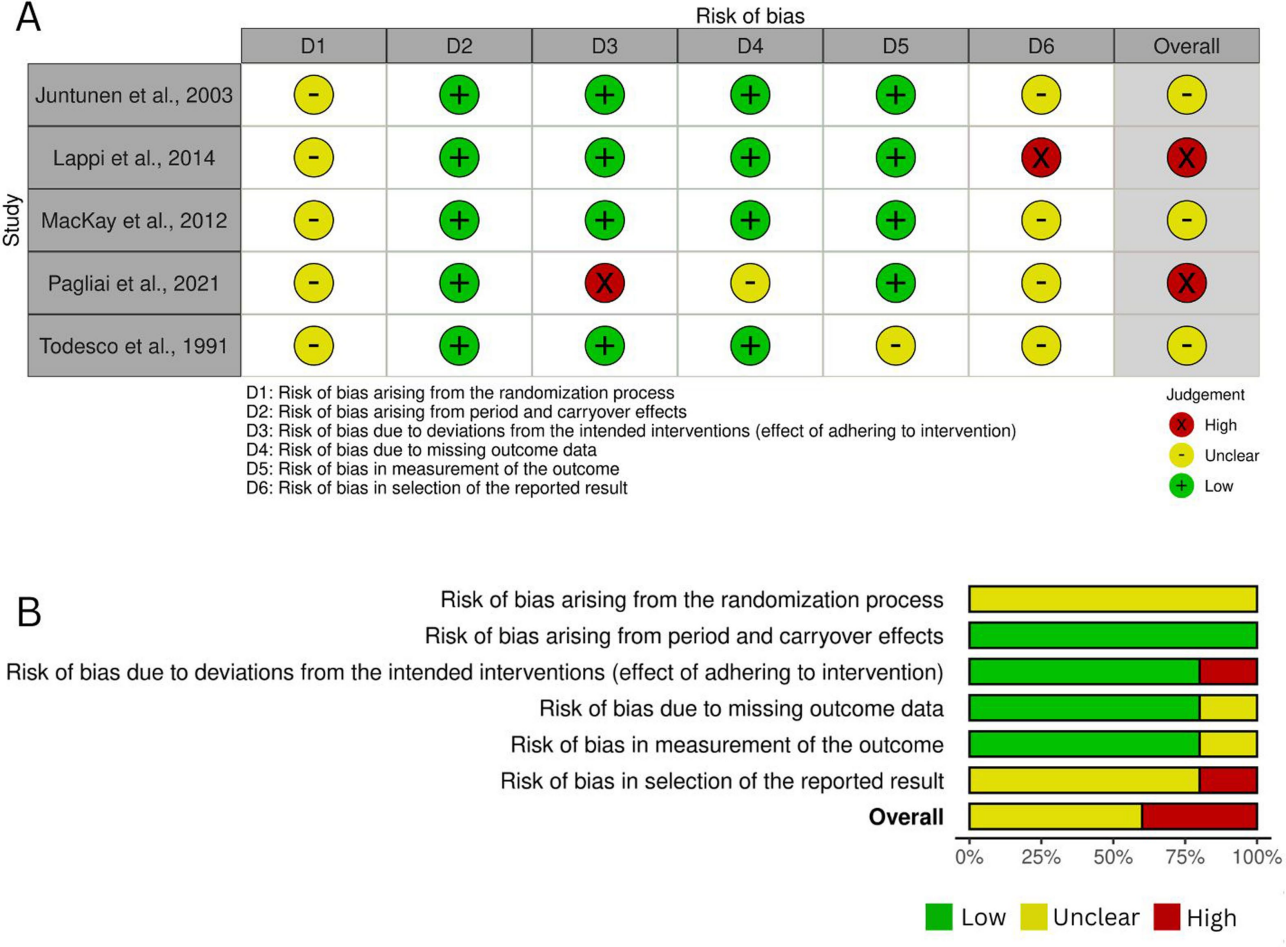
Risk of bias analysis of the randomized studies assessing the effect of fermented bread consumption on glycaemia. **(A)** Results for each study. **(B)** Summary per domain of risk.

For the 26 studies using a randomized controlled trial design, we assessed the risk of bias using the Cochrane RoB2 tool. Only two (7.7%) were judged to have a low risk of bias across all domains (26, 47). In contrast, five studies (19.2%) were judged to have a high overall risk of bias. The reasons were mainly deviations from intended interventions (27, 29, 49), selective reporting of results (68), or both (63). The majority of studies (19, 73.1%) were classified as having “*some concerns*” (Figures 2–4). For our first specific question on the effect of the type of fermentation, all studies showed a low risk of bias in measurement outcomes (Figure 2), whereas for the general effect of fermentation, the strength of included studies concerned missing outcome data, as all were assessed with a low risk of bias in this domain (Figure 3).

Overall, the most frequent concern was selective reporting, due to the absence of a publicly available protocol or a pre-specified statistical analysis plan. Due to the crossover design, some issues were identified in domains related to randomization, reporting, control of carryover effects, and the adequacy/appropriate choice of the control condition. In addition, some studies also raised concerns about missing outcome data (27, 64) and the measurement of outcomes (35, 37, 38, 62).

Therefore, details on the planned study including all parameters to be measured and all planned statistical analyses should be provided in the previously published study protocol. Moreover, datasets of the results should be published along with the manuscript with clear reporting on final statistical analyses (as Supplementary material or in repositories) to facilitate comparisons across studies and allow reuse of the data (e.g., for meta-analysis).

#### 3.3.4 Evaluating the certainty of the evidence from human studies

Considering the number of identified relevant studies (12), their risk of bias (majority with some concerns) and the consistency of effects (10 out of 12 showing a protective effect on glycaemia, 5 out of 7 on insulinaemia) we estimate overall level of the evidence for the effect of the type of fermentation as “low” for a beneficial effect of sourdough fermentation compared to yeast fermentation on postprandial glycaemia parameters. For the general effect of fermentation on postprandial glycaemia, due to a low consistency of observed effects, even if 10 human studies were found, most of them with only some concerns of risk of bias, we estimate the evidence as “very low”. The number of studies for subgroups of control products (unfermented breads, pasta, porridge/kernels) was insufficient to reach a higher level of evidence, even though the effects observed within each subgroup were more consistent.

Finally, we found only a few studies that contributed evidence on the overall effect of bread consumption on long term glycaemia/insulinaemia parameters. Most had some concerns for risk of bias and highly inconsistent effects. Therefore, we conclude to a “very low” evidence for a general effect on bread consumption on glycaemia/insulinaemia in over-weeks interventions. Still, for this specific question, the identified studies did not use an appropriate design to address our research question, as discussed above.

### 3.4 Biological plausibility: bioavailability and mechanism of action

**Figure 5:**
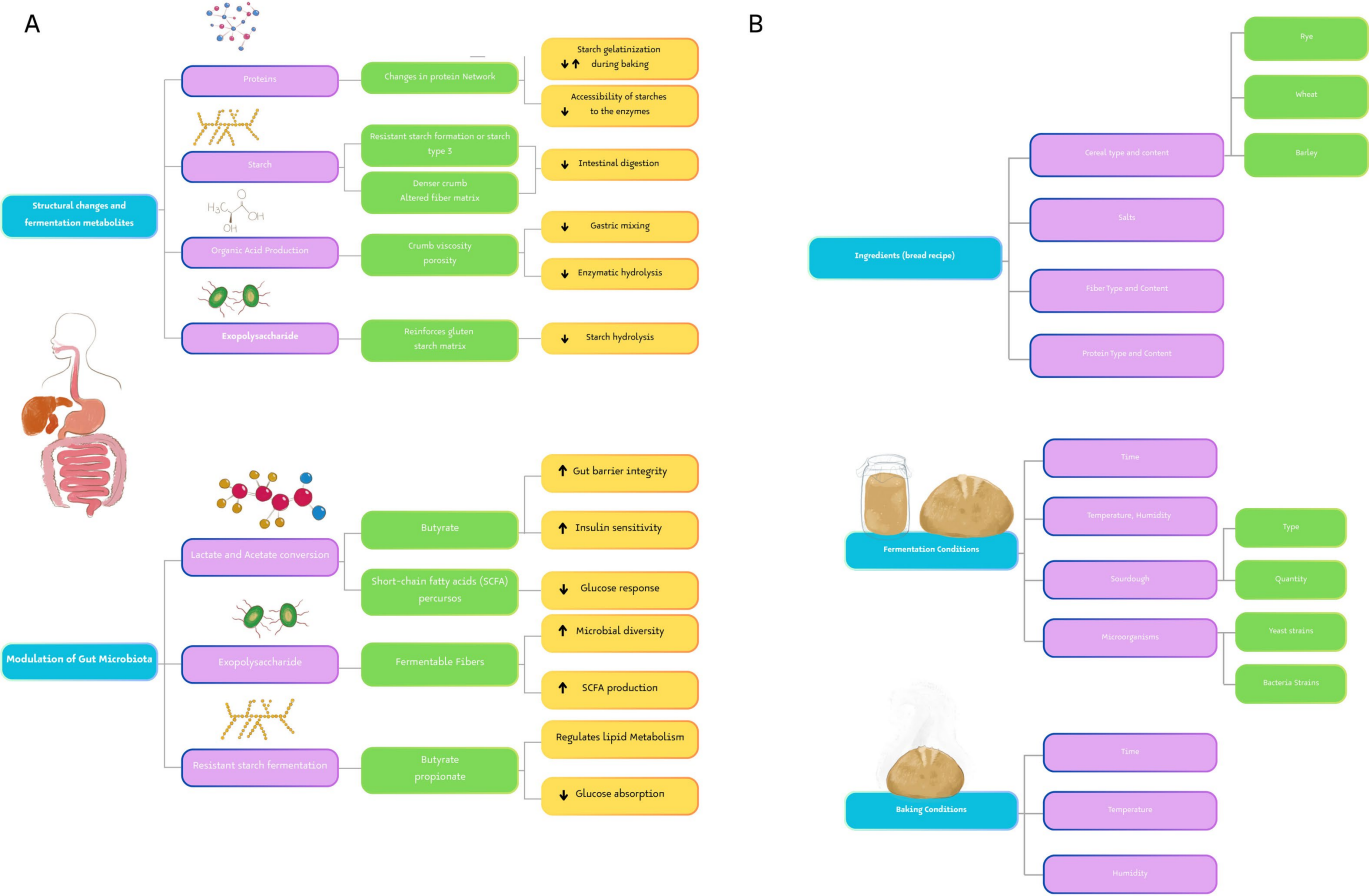
Overview of possible mechanisms of action **(A)** Main pathways behind the glycaemic effects of fermented breads. **(B)** Further factors possibly influencing glycaemic effects of fermented breads.

The potential role of commonly consumed fermented cereal-based products, such as bread, in glycaemic regulation is increasingly relevant to public health, given the T2D pandemic. While clinical trials in humans have provided supportive evidence for reduced postprandial glucose levels after the consumption of sourdough or lactic-acid-fermented breads, *in vitro* and *in vivo* studies have helped to define/uncover the possible mechanisms that contribute to this process, which can be grouped in (i) changes in bread structure that reduce the accessibility of starch to digestive enzymes, slowing glucose release, (ii) the production of organic acids and other bioactive compounds during fermentation, which lower bread pH and may slow gastric emptying and starch digestion and (iii) the potential modulation of gut microbiota and its metabolites (Figure 5).

#### 3.4.1 Bread structure and starch accessibility

Fermentation chemically and structurally modifies the starch and protein content of bread, influencing digestion kinetics and postprandial glucose dynamics (Figure 5A). Several *in vitro* and rodent studies have reported that sourdough fermentation can increase the proportion of resistant starch fractions, which are less accessible to enzymatic hydrolysis and could therefore contribute to a lower glycaemic potential (69–71). However, this relationship has not been consistently confirmed in the human studies included in the present review, and the extent of resistant starch formation appears to depend strongly on the flour type, fermentation conditions and baking conditions. Furthermore, while gelatinization during baking typically increases starch digestibility, other processes like fermentation and subsequent cooling promote the formation of resistant starch type 3 (retrograded starch), which further resists enzymatic hydrolysis and thereby can contribute to lower glycaemic potential (72). Moreover, bread fermented with LAB exhibits a denser crumb structure and altered fiber matrix, both of which are physical properties that reduce enzymatic access to starch substrates *in vitro* (71).

It has been reported that fermentation type and baking conditions also affect postprandial glycaemic and insulinemic responses by altering gastric emptying rates, starch digestibility, and nutrient bioavailability (27, 31). Specifically, sourdough fermentation has been associated with changes in the composition and functionality of dietary fiber, the release of bound phenolics, an increase in organic acid content, and reductions in the amount of simple carbohydrates and starch digestibility. All of these outcomes may delay gastric emptying and affect glucose metabolism (27, 33, 43).

#### 3.4.2 Organic acids from fermentation reduce pH and delay starch digestion

Additionally, several *in vitro* models of human digestion have shown that organic acids produced during fermentation, particularly lactic and acetic acids, slow starch hydrolysis by inhibiting amylase activity and reducing glucose release (Figure 5A) (65, 73, 74). Besides their effects on carbohydrate digestibility, organic acids produced by fermentation, including several short-chain fatty acids (SCFAs), can affect gastric emptying and regulate appetite. For example, acetate has been shown to cross the blood–brain barrier and activate hypothalamic pathways that regulate appetite and energy expenditure (75). This leads to increased satiety hormone levels, reduced food intake, and slower gastric emptying, contributing to more gradual postprandial glucose rises. In an older rodent study, lactic acid was also found to modulate postprandial plasma glucose concentration through delayed gastric emptying due to increased secretin and cholecystokinin gut release (76, 77).

Fermentation not only changes the carbohydrate fraction of the cereal but also alters its nutritional profile. It increases protein digestibility by degrading proteins through cereal proteases, which are activated by the organic acids produced by LAB and yeast (13). This can lead to the formation of bioactive peptides such as ACE-inhibitory peptides, whose primary function is to help lower blood pressure, but they may also influence glucose metabolism by improving insulin sensitivity and glucose uptake and reducing oxidative stress and inflammation, both of which are implicated in insulin resistance (78) and kokumi peptides, with anti- inflammatory and hypoglycaemic effects (79). Other products of fermentation include free amino acids, some of which are precursors to bioactive compounds such as 4-hydroxyphenyllactic acid, 2-hydroxy-isocaproic acid, vanillic acid, and 3-phenyllactic acid or *γ*-aminobutyric acid, which might also impact diabetes risk through their antioxidant activities (80).

#### 3.4.3 Modulation of gut microbiota and its metabolites

The final mechanism that has been proposed to participate in the effect of sourdough bread in glucose regulation is the gut microbiota and its metabolites that are affected by dietary exposure to sourdough bread (Figure 5A). Both undigested carbohydrates from cereals and exopolysaccharides formed by the LAB during fermentation (81) can be an energy source for the gut microbiota, which can metabolize them into SCFA. *In vitro* fermentation experiments using human fecal inocula have demonstrated that sourdough and LAB-fermented breads yield higher SCFA concentrations compared to conventional breads, with coarse wholemeal rye and wheat-rye breads showing particularly favorable SCFA production patterns ((82, 83). These SCFAs, especially butyrate and propionate, have been shown in cell culture to activate AMP-activated protein kinase (AMPK) in skeletal muscle, enhancing GLUT4 translocation and glucose uptake. They also stimulate the secretion of incretin hormones such as GLP- 1 and Peptide YY (PYY) from enteroendocrine cells (84) through activation of free fatty acid receptors (FFAR2/3). FFAR2/3, found also in vascular tissue, adipose tissue, pancreas, and other organs, are associated with improved glucose homeostasis, enhanced insulin sensitivity, and modulation of blood pressure. This association has been largely established through gain-of-function (overexpression) or loss-of-function (knockout) in transgenic rodents or cell models, often with SCFA administration to activate the receptors, showing that FFAR2/3 play beneficial roles in glucose and insulin regulation (85, 86). Activation of these receptors has also been linked to elevated plasma leptin and reduced non-esterified fatty acid levels (63, 64, 77). Moreover, improved endothelial function, partially mediated by SCFAs and by stimulation of nitric oxide production, is closely linked to better vascular insulin sensitivity, which facilitates glucose uptake by tissues (87).

The impact of sourdough on the gut microbiota composition and metabolites has been investigated principally using *in vitro* systems to simulate food-microbiome interactions (88), with several animal studies, some investigating fermented bread (89, 90) while others focus on the effects of isolated sourdough cultures (91), but there is little evidence from human studies (32). Despite the potential mechanisms by which fermented bread could affect the gut microbiota to improve glucose regulation, *in vivo* data is inconsistent. Shifts in specific microbial groups were observed for two studies that supplemented mice (90) and rats (89) with fermented bread (sourdough and yeast-fermented) compared to the control group, but the effects on glycemia, both pre- and post-meal, were minimal for all groups.

Consistent changes in the microbiota were observed in sourdough- and yeast-fermented bread interventions compared to the control in both studies, but the taxa differed between the studies. Moreover, significant differences were observed in both studies for the *Mucispirillum* genus comparing the two types of fermented breads but the genus was higher for mice in the sourdough group than those in the yeast-fermented bread group in the mice study mice (90), while reduced for rats in the sourdough group relative to the yeast-fermented bread group (89). Rats fed with diets incorporating sourdough-fermented breads showed significantly improved postprandial glucose profiles compared to those consuming conventionally leavened bread (92). The study reported reductions in glycemia and insulinemia, as well as lower levels of pro-inflammatory cytokines, suggesting broader metabolic benefit associated with the consumption of sourdough-fermented bread. These results highlight that sourdough fermentation may not only modulate glycaemic response but also exert anti-inflammatory effects and improve overall metabolic health in animal models.

Similar to the described animal studies, effects of sourdough bread on the gut microbiota have been observed in human studies, but not in parallel to changes in glycaemia. Notably, Korem et al. (32) found significant increases after white bread consumption, relative to sourdough bread consumption, on two butyrate producers (*Eubacterium ventriosum* species and the *Anaerostipes* genus), in a short-term crossover intervention. High inter-individual variability in glucose response to different breads was observed in this study, but, surprisingly, the direction of the response (higher in sourdough or yeast-fermented bread) could be reliably predicted from features of the baseline microbiome data. These findings indicate that not only can the gut microbiome be altered by fermented breads to affect glucose homeostasis but the current gut microbiota may play a critical role in mediating the response to different bread interventions.

The role of SCFA in glucose regulation is widely investigated. For example, it has been reported that propionate suppresses gluconeogenesis in hepatocytes by inhibiting phosphoenolpyruvate carboxykinase (PEPCK), while butyrate enhances glycogen synthesis, both of which contribute to improved postprandial glucose regulation (93, 94). Moreover, in isolated pancreatic islet cells from rodents, butyrate showed the most pronounced effect on inducing insulin secretion compared with other SCFA tested (95).

#### 3.4.4 Conclusions on mechanisms of action

Together, *in vitro* and *in vivo* animal studies provide mechanistic evidence that supports and complements clinical research on the glucose-lowering potential of fermented breads. By modulating the rate and extent of starch digestion and influencing host metabolic and inflammatory pathways, fermented breads, especially those produced using traditional sourdough or lactic acid, could be a promising dietary intervention for glycaemic control. However, the impact of fermented bread on glycaemia appears to both influence and be influenced by additional individual factors, such as the gut microbiome composition itself, which should be considered in interventions assessing the role of fermented bread on glycaemia (96). Additionally, the role of the upper intestinal tract in glycaemic response remains unclear. Different food structures have been shown to elicit distinct postprandial gut hormone responses and satiety via GLP-1 and PYY (97). This suggests that dietary effects might be more complex and interconnected than initially suspected and that it would require integrating person-specific factors with food characteristics. Where feasible, inclusion of complementary measurements *in vivo* (e.g., gastric motility for postprandial studies or gut microbiota parameters for long term studies) and/or *in vitro* (e.g., enzymatic hydrolysis) should be considered to support interpretation of clinical outcomes and directly link proposed mechanisms observed *in vitro* and evidence from human studies.

### 3.5 Safety of fermented breads

None of the studies included in this review reported major safety concerns related to fermented bread intake. Mild, non-serious adverse effects were occasionally observed, such as the flatulence noted by Eelderink et al. (45), which was not linked to any specific test meal and was deemed clinically irrelevant. Similarly, Rave et al. (34) recorded 30 gastrointestinal events during their intervention (15 during wholegrain treatment, 13 with meal replacement, and two during the washout phase), though these effects were attributed to bread ingredients rather than the fermentation process. Three participants experienced transient constipation early in the wholegrain treatment, which resolved without consequence, and no such effects were reported during the meal replacement phase. Available evidence does not indicate any safety issues associated with bread consumption.

From a technological perspective, sourdough fermentation lowers the dough pH and produces organic acids, creating an environment that inhibits the growth of spoilage and pathogenic microorganisms (12). Moreover, fermentation can reduce levels of potentially harmful compounds such as mycotoxins and acrylamide precursors, further improving product safety (98). Furthermore, sourdough fermentation can mitigate acrylamide formation by lowering dough pH, which inhibits the Maillard reaction, and by promoting the activity of LAB and yeast that degrade free asparagine, a key acrylamide precursor (99, 100). Studies have demonstrated that the use of specific sourdough starters or extended fermentation time can significantly reduce acrylamide concentrations in bread compared with yeast- leavened controls, with reductions of up to 70% reported in some formulations (101). Nevertheless, as with other fermented foods, inadequate process control or inappropriate storage conditions may permit the accumulation of biogenic amines or survival of undesirable microbes, highlighting the importance of maintaining good manufacturing practices and controlled fermentation parameters (102).

Other safety issues should be considered for cereal-based foods, particularly wheat products, including their potential allergenicity and inflammatory effects. The extent to which fermentation, particularly sourdough, can influence these risks was not the focus of our study and is reviewed elsewhere (14).

### 3.6 Characterization of the relationship between consumption of fermented bread and functional effects

Fermented breads, particularly those using sourdough starters with LAB and yeasts, have been studied widely for their potential to modulate postprandial glycaemic and insulinaemic responses. Human dietary intervention studies mostly show that sourdough bread tends to slightly reduce postprandial glucose and/or insulin levels compared to conventional yeast-leavened bread. Differences in the outcome effects can be due to differences in study design (duration, population) but also to the breads used, formulation and production varying greatly between studies, if at all reported. Despite these discrepancies, sourdough bread is generally associated with delayed gastric emptying, reduced starch hydrolysis, and lower availability of quickly digestible sugars. These effects are primarily observed in short-term postprandial settings, while longer-term effects remain unclear due to study design limitations. A tendency toward lower glycaemic and/or insulinemic responses after consuming sourdough bread has been demonstrated in acute intervention studies (26, 48, 64, 65). The mechanisms proposed for these effects include acid-induced delay of gastric emptying and inhibition of amylolytic enzymes, as demonstrated by studies with 13C-octanoic acid labeling (28, 103).

According to some studies, such as those conducted by Chatonidi et al. (28) and Liljeberg et al. (48), breads with similar acid contents, whether from fermentation or direct acid addition, produced comparable metabolic responses, highlighting the role of fermentation by-products. However, only part of the selected studies analyzed their test bread for acidity-related parameters; some reported only pH or a single specific organic acid. This lack of standardized information on the bread chemical characteristics impairs a detailed comparison of studies and reduces the strength of evidence on the relationship between these parameters and effects on glycaemia response.

Beyond the reductions in glucose and insulin peaks, some studies (28, 68, 103), reported lower C- peptide levels and altered insulin dynamics, suggesting potential improvements in insulin sensitivity underlying the glycaemia changes. However, not all studies have reported consistent outcomes. For example, Dall’Asta et al. (44) found no significant differences in metabolic outcomes, potentially due to variations in flour type, fermentation duration, or microbial strains involved. The lack of reporting on production conditions as well as on the microbial composition of sourdough used or in bread dough after fermentation in several studies, however, again impairs comparisons between studies and a strong conclusion on its exact effect on observed outcomes.

Dose–response effects of bread consumption were not directly explored in the identified studies. Novotni et al. (26) demonstrated a non-linear dose–response, where moderate amounts of sourdough ferment used for bread preparation reduced GI more effectively than bread with higher doses of ferment, possibly due to changes in food matrix viscosity, showing the importance of the structure characterization of tested breads. Still, structural characterization was absent from most of the selected studies, even if some studies showed a possible role of structural parameters such as starch gelatinization or compact amylopectin-amylose networks (43, 54). Liljeberg et al. (48) found that the presence of lactic and propionic acids at defined concentrations significantly lowered glucose and insulin responses, mimicking the effects of sourdough fermentation.

Despite the relatively consistent findings in acute interventions, only a few studies were identified that assess long-term glycaemia/insulinaemia effects, making it difficult to extrapolate findings to habitual consumption patterns. Moreover, the findings from these studies were inconsistent and poorly controlled for factors relevant to our research question. The effects observed in acute interventions were mostly of a small magnitude and thus may not translate to a significant impact on the fasting measures of glycaemia and insulinaemia in relatively short exposures used in the ‘long-term’ intervention studies that we identified. Moreover, while dynamic and variable responses are observed in postprandial glycaemia measures, fasting measures of glycaemia are tightly regulated in healthy individuals, reflecting a physiological homeostatic control that should not be perturbed by short-term dietary intervention. Observational studies that consider changes over years of consumption are better suited to assess the long-term impact of fermented breads. However, we could not identify any such study that addresses our blood-measurement-based outcomes of interest while also providing sufficient granularity in the dietary assessments to permit such investigation. Bread formulations also vary significantly across studies, where described, with different flour types, fermentation times, pH levels, and inclusion of additives (e.g., gluten, fat, salt), which makes direct comparisons and meta-analysis difficult (Figure 5B).

A further consideration regarding the available evidence is its geographical concentration: 21 of the 27 included studies were conducted in Europe (eleven in Nordic countries), and only one study was conducted in Asia (China), with no studies from South or Southeast Asia or from Africa meeting our inclusion criteria. This distribution is broadly aligned with the role of bread as a major dietary staple: bread intake is high in many European and Middle-Eastern countries, where wheat- and rye-based breads (including traditional sourdough) are central to diets, whereas in most of Asia, rice is the dominant cereal, and in much of sub-Saharan Africa, coarse grains like maize, sorghum and millet (104, 105). The geographical concentration is therefore aligned with the populations for which fermented bread is an important source of carbohydrates. Studies in non-European populations are desirable, especially now that its consumption is rising (106). Nevertheless, the limited number of such studies is unlikely to substantially modify the overall direction of the evidence reviewed here.

The inconsistent findings cannot be explained solely by methodological heterogeneity. Several well-conducted studies did not demonstrate significant metabolic benefits, suggesting that the effects of fermentation are modest and depend on factors such as bread formulation, fermentation conditions, and participants’ metabolic status. Moreover, mechanisms demonstrated *in vitro* or in animal models may not consistently translate into measurable clinical benefits in healthy individuals.

## 4 Conclusion

We identified well-studied food characteristics and proposed mechanisms of action sustaining a biological plausibility, as well as low evidence, assessed from the human studies meeting our inclusion criteria, for a modest reduction of postprandial glycaemic and/or insulinaemic responses after sourdough bread compared to yeast bread. Based on this information the available evidence remains “neither convincing nor sufficient” to fulfill the requirements for scientific substantiation according to EFSA standards. Particularly the absence of observational studies, lack of dose–response trials, and insufficient long-term data limits the interpretation of the available evidence.

In the case of the effect of fermented bread intake in general (compared to non-fermented cereal products or over a longer intervention period), there is currently “no or only very low low” evidence for a positive effect on glycaemia/insulinaemia homeostasis. This is mainly due to inconsistency of observed effects or absence of well controlled long-term studies.

It should be noted that our conclusions reflect the small number of studies meeting our inclusion criteria, their heterogeneity in design and predominantly small sample sizes, and their generally small sample sizes, including a strong geographical concentration in European populations, particularly in Nordic countries. The external validity of the available evidence is therefore limited. Moreover, many interventions used highly standardized experimental breads that may not reflect commercially available products or traditional bread-making practices. Differences in bread formulation, fermentation processes, and dietary habits across populations further limit the generalizability of the findings. Therefore, larger multicentre studies using commercially relevant bread products and more diverse populations are warranted.

To advance the scientific substantiation of health benefits associated with fermented bread, future studies should focus on long-term (≥4 weeks) randomized controlled trials with well-characterized breads. We recommend including structural characteristics (measured with validated methods) and for sourdough bread microbial composition of sourdough/dough as well as pH and organic acids content. We also recommend to describe in detail bread formulations and preparations (including time of fermentation). An adequate number of participants to observe a change in the selected outcome should be included. The investigation of mechanistic endpoints (e.g., gastric motility, enzymatic hydrolysis, gut microbiota) in such controlled studies could help understand the disparity between studies and help understand the pathways involved behind observed effects. Finally, data from observational, prospective cohort studies that assess real-world outcomes, such as T2D incidence, should be used to determine whether the observed short-term metabolic effects in healthy individuals translate into meaningful population-level health benefits.

## Supplementary material

SUPPLEMENTARY FILE 1 PRISMA checklist.

SUPPLEMENTARY FILE 2 Table on the strings used for literature search.

SUPPLEMENTARY FILE 3 Table on the characteristics of the studies, fermented bread used and main results on glycaemia/insulinaemia outcomes for the impact of the type of fermentation on the glycaemic effects of bread.

SUPPLEMENTARY FILE 4 Table on the characteristics of the studies, bread and control used and main results on glycaemia/insulinaemia outcomes for the general impact of fermentation on the glycaemic effects of bread.

SUPPLEMENTARY FILE 5 Table on the characteristics of the studies, fermented bread used and main results on glycaemia/insulinaemia outcomes for effect of fermented bread consumption on glycaemia.
